# Epidemiology of proximal humerus fractures

**DOI:** 10.1186/s13018-021-02551-x

**Published:** 2021-06-22

**Authors:** Sandra Iglesias-Rodríguez, Diego Matías Domínguez-Prado, Alejandro García-Reza, Daniel Fernández-Fernández, Elena Pérez-Alfonso, Javier García-Piñeiro, Manuel Castro-Menéndez

**Affiliations:** Servicio de Cirugía Ortopédica y Traumatología, Complejo Hospitalario Universitario de Vigo (Pontevedra), Estrada de Clara Campoamor, 341, 36213, Vigo, Pontevedra, Spain

## Introduction

Proximal humerus fractures (PHF) are very common and a serious health problem [1]. They are the seventh most frequent fractures in adults [2]. Their prevalence varies from 4 to 10% of all fractures, according to several studies conducted in different populations [3]. Their treatment is sometimes controversial and some cases may be technically challenging. The incidence rate of these fractures shows considerable variation depending on the geographical area and the year of the study [4, 5]. This rate has been suggested to be increasing along with the increase of elderly patients [6–8].

In order to plan health prevention protocols and to establish economic models in this field, up-to-date epidemiological data are necessary [1, 9]. Although several epidemiological studies on PHF have been conducted in Europe [1, 4–7, 9–13], few have included both inpatients and outpatients [1, 3, 4, 9]. No recent study of this kind has been found in the Iberian Peninsula nor in other southern European countries.

The aim of this study is to analyse PHFs from an epidemiologic and descriptive perspective, including the specific characteristics of the patients who suffer them, their incidence rate, the type of fracture and the methods used for treatment, and to compare these data with the available records of other countries.

## Material and methods

Vigo is a Spanish city located in the northwest of the Iberian Peninsula, in the province of Pontevedra, Autonomous Community of Galicia. With a population of 295,364 inhabitants, it is the most populated city of Galicia and the fourteenth of Spain. It is also the urban core of a metropolitan area which comprises other fourteen municipalities, with a total population of 480,525 inhabitants. The Vigo University Hospital Complex (CHUVI) sees patients living in this metropolitan area.

A retrospective, observational study was conducted including all patients over 18 years of age who were admitted into the CHUVI A&E department and diagnosed with PHF between January 1, 2016, and December 31, 2018.

Data were recorded as follows. All patients who were admitted into the emergency department in CHUVI and needed medical care provided by Trauma and Orthopedics (T&O) were coded and divided into two groups: those with upper-limb involvement and those with lower-limb/spine involvement. The visits with upper-limb pathology recorded were subsequently revised and patients diagnosed with PHF were identified. Patients meeting these requirements but from outside the hospital’s sphere of influence were excluded from the study.

A standard imaging-test protocol for shoulder injuries was originally performed to all patients with suspected PHF. The protocol consists of an anterior-posterior x-ray (with the tangential central ray onto the glenoid surface) and a Velpeau axillary projection (with the patient’s arm in internal rotation and the ray from cranial to caudal with the patient leaning backwards). In cases where a complex fracture was identified in the x-ray, a computed tomography (CT) was requested.

The following data were recorded from our patient selection: gender, age, laterality, type of trauma (high-energy traumas which included sports accidents, traffic accidents, falls from heights of more than 2 m, or low-energy trauma which included fall from standing height or syncope), season of the year when the fracture happened, pre-existing comorbidities (cardiovascular, neurological and/or psychiatric illness, alcohol abuse and smoking, confirmed diagnosis of osteoporosis, diabetes mellitus, obesity, malignancies, rheumatological diseases, endocrine diseases, and other metabolic disorders), and type of treatment for each PHF.

We reviewed the x-ray and CT scan of all patients in order to classify them according to the three AO-OTA [14] types (A-C), its nine groups (A1-C3), and the number of PHF parts according to the Neer classification [15] (types I-V).

Data collection was carried out by five orthopaedic surgeons and further analysed by an independent observer.

The total number and the gender and age distributions of the population at risk for each year of the study period were obtained from the official CHUVI website (available at: https://xxivigo.sergas.gal/Paxinas/web.aspx?tipo=paxtxt&idLista=3&idContido=272&migtab=272&idTax=850)

The incidence rate is a measure that reflects the risk of developing a new disease over a specified time period [16]. In this particular case, it measures a person’s risk of suffering a PHF within the period of 1 year. The incidence rate is calculated as follows [17]:

$$ \mathrm{Incidence}\ \mathrm{rate}=\frac{\mathrm{Number}\ \mathrm{of}\ \mathrm{events}\ \mathrm{occurring}\ \mathrm{during}\ a\ \mathrm{specific}\ \mathrm{time}\ \mathrm{period}}{\mathrm{Population}\ \mathrm{at}\ \mathrm{risk}\ \mathrm{over}\ a\ \mathrm{specified}\ \mathrm{time}\ \mathrm{period}}\times {10}^{\mathrm{n}} $$

In this study, the number of new events was the annual average number of PHF cases that occurred between 2016 and 2018. The population at risk was estimated using the median interval of the population during the specified time period (2016–2018). The gross incidence rate and gender-specific and age-specific incidence rates were calculated using age ranges per 100,000 inhabitants per year.

The study was approved by the Hospital/Regional Ethics Committee*.* The researchers conducted the study in accordance with the principles of the Declaration of Helsinki. The study was developed according to the protocol and in compliance with the standards of good clinical practice, as described in the guidelines of the International Council for Harmonisation (ICH) on good clinical practice.

### Statistical analysis

The statistical analysis was carried out using the IBM software SPSS Statistics® v22. Continuous variables were described by using the mean, the standard deviation, and minimum and maximum values. Discrete variables were described using frequency distribution and percentages.

In the bivariate analysis, the Student-Fisher t test was used for continuous variables and chi-squared test was used for categorical variables. Pearson’s and Spearman’s correlation coefficients were used for the association between variables. Differences were considered significant if *p* < 0.05.

## Results

In the period under review, there were 638 fractures (192 in 2016, 213 in 2017, and 233 in 2018). There were 7 patients (5 women and 2 men) who suffered two PHF but in periods of more than 6 months apart (4 patients in the contralateral shoulder and 3 in the ipsilateral shoulder), so they were considered as independent cases. In no case, a bilateral PHF was registered in the same visit to the emergency department.

### Age and gender

The sample included 495 women (77.6%) and 143 men (22.4%), with a ratio of 3.4:1. By subdividing patients into groups sorted by decade, there was a female to male ratio of 1:1 up to 50 years old, which evolved to 4:1 above that age. A statistically significant association was found between the variables age and gender (*p* < 0.001).

**Fig. 1:**
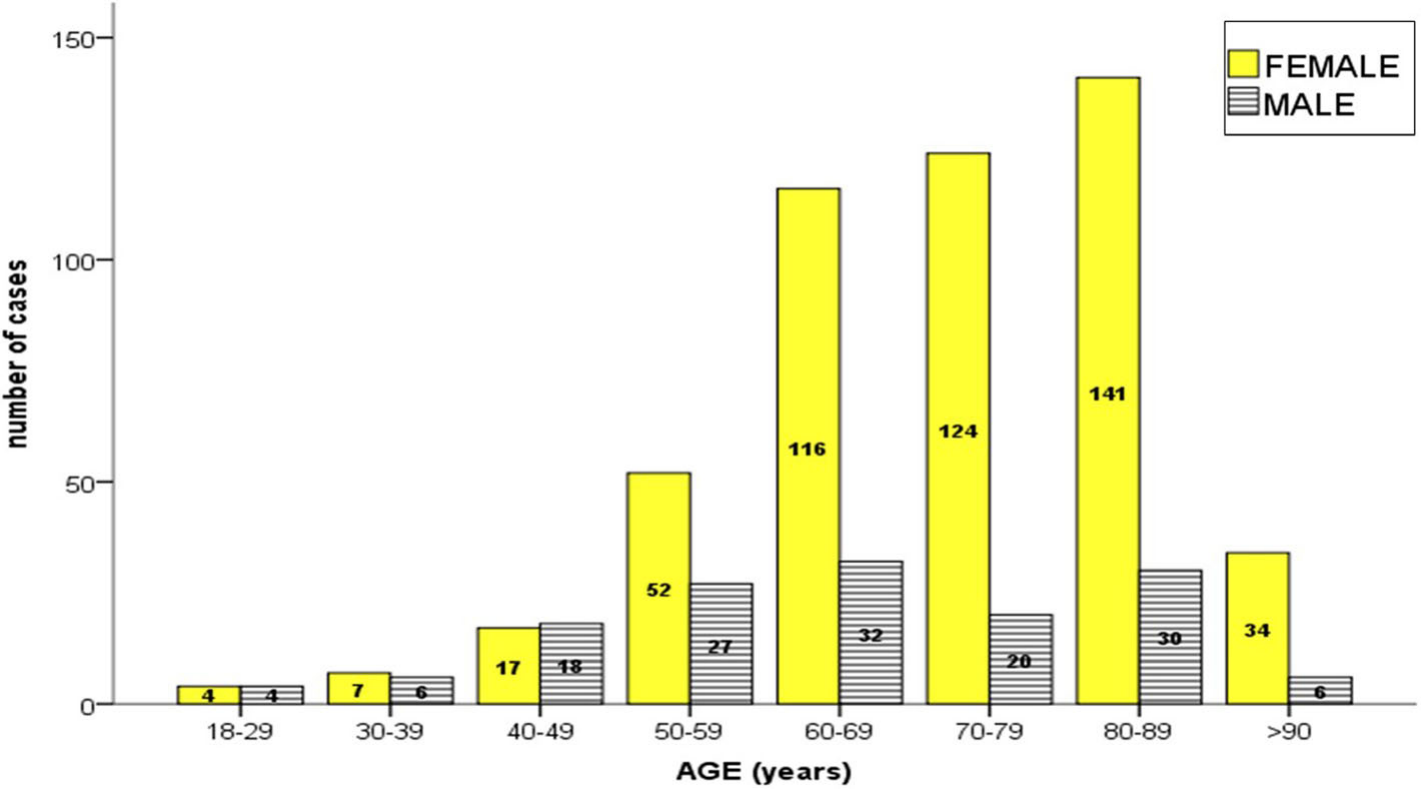
Number of cases according to age

Average patient age was 70.4 ± 12.2 years (minimum 18 and maximum 101 years). By subdividing into groups by 5-year periods (Fig. 1), a progressive increase was observed up to 60 years old, which remained similar from this decade up to 90 years old. Beyond the age of 90, the percentage decreased again. Octogenarians (80 to 89 years old) were the largest group (26.8%). The cumulative percentage of patients aged 60 or older accounted for 78.8% of the sample.

### Affected side

The right side was affected 330 times (258 women and 72 men), which amounts to 51.7%, while the left was involved 308 times (48.3%). There was no statistically significant association between laterality and gender (*p* = 0.7).

### Injury mechanism

Only in 3.6% of cases (23 patients) was there high-energy trauma (13 women and 10 men), while the rest (96.4%) were low energy. A statistically significant association between the type of trauma and age was observed, grouped by 5-year periods (*p* < 0.001). Most high-energy injuries (73.9%) occurred in patients under 60, while only 20.7% of low-energy injuries were recorded in this age range. We have also found a statistically significant association between the type of trauma and the type of treatment provided, since 43.5% of high-energy fractures were operated on, while only 18.7% of low-energy injuries needed surgery (*p* < 0.001).

### Seasonal variation

The season when there were more fractures was Autumn, with 179 fractures (28.14%), followed by Spring with 166 cases (26%), with Winter with the lowest number of fractures recorded (134 fractures, 21%). No statistically significant difference was found between them (*p* = 0.8). There was no statistically significant association between the season when the fracture occurred and gender (*p* = 0.4), age (*p* = 0.3), type of trauma (*p* = 0.6), or with the AO-OTA [14] (*p* = 0.6) or Neer [15] (*p* = 0.1) classifications (Table 1).

**Table 1:** Patients of study divided by sex, age range, type of treatment, and mechanism of injury according to AO-OTA [14] and Neer [15] classifications. (y, years old; N, number of cases; % group, percentage cases within this AO-OTA group or NEER group classification; % age, percentage cases within this range of age)

				AO_OTA classification			All	Neer classification					All
				A	B	C		Non displaced	2-part	3-part	4-part	Fracture-dislocation	
**Female**		**< 50y**	N	26	4	1	31	17	11	2	1	0	31
			% age	83.9%	12.9%	3.2%	100%	54.8%	35.5%	6.5%	3.2%	0.0%	100%
			% group	11.1%	1.8%	2.6%	6.3%	14.8%	6.9%	1.2%	3.0%	0.0%	6.3%
		**> 50y**	N	208	219	37	464	98	149	159	32	26	464
			% age	44.8%	47.2%	8.0%	100%	21.1%	32.1%	34.3%	6.9%	5.6%	100%
			% group	88.9%	98.2%	97.4%	93.7%	85.2%	93.1%	98.8%	97.0%	100.0%	93.7%
**Male**		< 50y	N	21	7	1	29	13	8	5	1	2	29
			% age	72.4%	24.1%	3.4%	100%	44.8%	27.6%	17.2%	3.4%	6.9%	100%
			% group	26.3%	13.5%	9.1%	20.3%	32.5%	17.4%	13.9%	9.1%	20.0%	20.3%
		> 50y	N	59	45	10	114	27	38	31	10	8	114
			% age	51.8%	39.5%	8.8%	100%	23.7%	33.3%	27.2%	8.8%	7.0%	100%
			% group	73.8%	86.5%	90.9%	79.7%	67.5%	82.6%	86.1%	90.9%	80.0%	79.7%
**Treatment**	**Conservative**	< 50y	N	35	7	0	42	30	8	3	0	1	42
			% age	83.3%	16.7%	0.0%	100%	71.4%	19.0%	7.1%	0.0%	2.4%	100%
			% group	13.0%	3.0%	0.0%	8.2%	19.9%	4.9%	1.9%	0.0%	3.3%	8.2%
		**> 50y**	N	235	224	12	471	121	155	153	13	29	471
			% age	49.9%	47.6%	2.5%	100%	25.7%	32.9%	32.5%	2.8%	6.2%	100%
			% group	87.0%	97.0%	100%	91.8%	80.1%	95.1%	98.1%	100.0%	96.7%	91.8%
	**Surgery**	**< 50y**	N	12	4	2	18	0	11	4	2	1	18
			% age	66.7%	22.2%	11.1%	100%	0%	61.1%	22.2%	11.1%	5.6%	100%
			% group	27.3%	9.1%	5.4%	14.4%	0%	25.6%	9.8%	6.5%	16.7%	14.4%
		**> 50y**	N	32	40	35	107	4	32	37	29	5	107
			% age	29.9%	37.4%	32.7%	100%	3.7%	29.9%	34.6%	27.1%	4.7%	100%
			% group	72.7%	90.9%	94.6%	85.6%	100%	74.4%	90.2%	93.5%	83.3%	85.6%
**Mechanics**		**High energy**	N	15	6	2	23	5	9	5	2	2	23
			% Hihg E	65.2%	26.1%	8.7%	100%	21.7%	39.1%	21.7%	8.7%	8.7%	100%
			% group	4.8%	2.2%	4.1%	3.6%	3.2%	4.4%	2.5%	4.5%	5.6%	3.6%
		**Low energy**	N	299	269	47	615	150	197	192	42	34	615
			% Low E	48.6%	43.7%	7.6%	100%	24.4%	32.0%	31.2%	6.8%	5.5%	100%
			% group	95.2%	97.8%	95.9%	96.4%	96.8%	95.6%	97.5%	95.5%	94.4%	96.4%

### Comorbidities

**Fig. 2:**
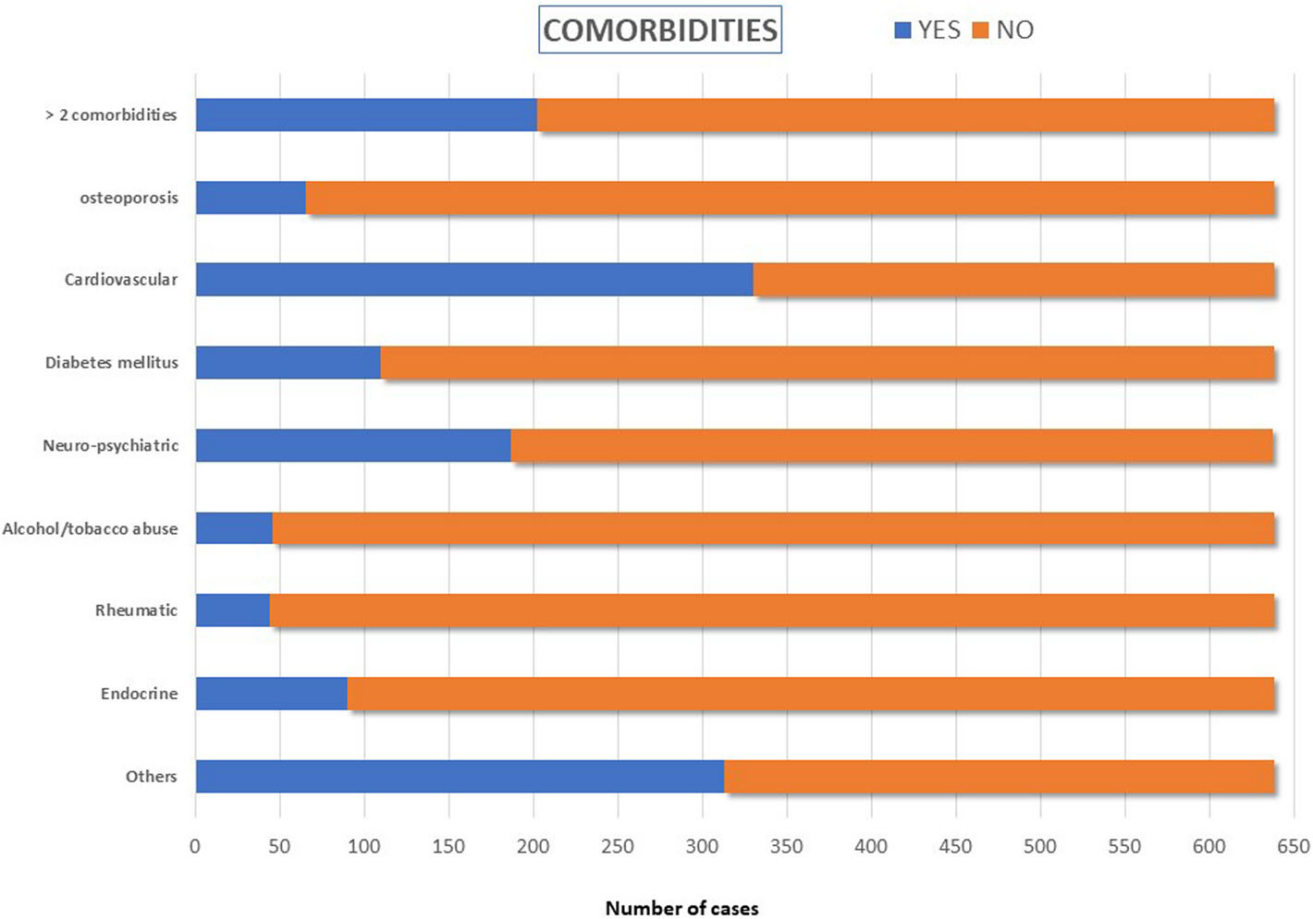
Comorbidities asocciated with fracture cases

A total of 112 (17.6%) had no comorbidity, 324 (50.8%) had one or two pathologies, and 202 (30.9%) had three or more. The most frequent pathology was cardiovascular disease which occurred in 51.7% of patients (330 cases) (Fig. 2). We found a statistically significant association between the presence of comorbidity and the age of patients (*p* < 0.001)—the patients over 65 years old represent 76.2% of those who have some kind of pathology and 82% of patients of the sample with multiple pathological problems. There is also a statistically significant association (*p* = 0.004) between the existence of pathology and gender, with women accounting for 80% of patients with some kind of pathology in our sample and 86.6% of patients with many diseases.

Only 10.2% of patients were under treatment for osteoporosis (64 women and 1 man). This factor was not significantly associated with the type of trauma (high or low energy) (*p* = 0.4) or with the treatment carried out (conservative or surgical) (*p* = 0.5).

### Fracture classification

According to the AO-OTA classification [14], 314 fractures were grouped into type A (49.2%), 275 into type B (43.1%) and 49 into type C (7.7%). According to this classification, subgroup B1 ranked first with 226 cases (34.4%), followed by A1 with 119 (18.1%) and A2 with 111 (17%). According to the Neer classification [15], there were 155 non-displaced or minimally displaced proximal humeral fractures (24.3%), 206 two-part displaced proximal humeral fractures (32.3%), 197 three-part fractures (30.9%), 44 four-part fractures (6.9%), and finally, in 36 cases, fracture was associated with a glenohumeral dislocation (5.6%). We have not found any statistically significant association between gender and the AO-OTA classification (*p* = 0.1) or the Neer classification (*p* = 0.8). The type of trauma did not show a statistically significant association with the AO-OTA classification (*p* = 0.2) or with the Neer classification (*p* = 0.9), as the diagnosis of osteoporosis did not show it either (*p* = 0.4 and *p* = 0.5 respectively). However, there was certainly a statistically significant association between these two PHF classifications and age grouped by decades (*p* = 0.02 for AO-OTA and *p* = 0.04 for Neer) and also with the type of treatment performed in each patient (*p* < 0.001 in both classifications) (Table 1).

### Type of treatment

A total of 125 patients (19.6%) underwent surgical treatment and the others were treated conservatively. Of the patients operated, 44 had a type-A fracture according to the AO-OTA classification [14] (35.2%), 44 other patients had type-B PHF, and 37 type C (29.6%). According to the NEER classification [15], 4 patients had a type-I fracture (all of these patients another ipsilateral fracture associated which required surgical treatment), 43 patients had a two-part fracture (20.9% of this group), 41 a three-part fracture (20.8% of the group), 31 a 4-part fracture (70.5% the group), and only 16.7% of fractures-dislocation (6 patients) were surgically treated (Table 1).

The most used surgical treatment was locking plates in 52 patients (41.6%), followed by reverse total shoulder arthroplasty in 36 cases (28.8%) and intramedullary nailing in 16 cases (12.8%). The most used fixation method in AO-OTA type B PHFs were locking plates (59.1% of the type B PHF), whereas in type C arthroplasties were the most common procedure (67.6% of the type C) and in type A both locking plates and intramedullary nailing ranked first (31.8% both). As regards the NEER classification, locking plates were used on three occasions and an intramedullary nail once to treat minimally displaced fractures; the most used treatment in two-part fractures were both locking plates and intramedullary nails (30% each for this PHF type), locking plates for 3-part fractures (56% of 3-part fractures), and reverse total shoulder arthroplasty for 4-part fractures (67.7%) of this group. Fractures-dislocations were treated surgically with locking plates on two occasions and with arthroplasty on three. Cannulated screws were also used as a fixation method in 13 cases (10 cases of 2-part fractures affecting the greater tubercle and 3 cases of 3-part fractures) and Kirschner needles in 8 cases (3 cases of 2-part fractures and 5 of 3-part fractures). We found a statistically significant association between the NEER classification and the method of osteosynthesis used (*p* < 0.001).

### Incidence

The risk population over 18 years old of the CHUVI healthcare area was estimated at 353,403 people/year between January 1, 2016, and December 31, 2018. There were 168,663 men and 184,739 women per year when disaggregating gender.

**Fig. 3:**
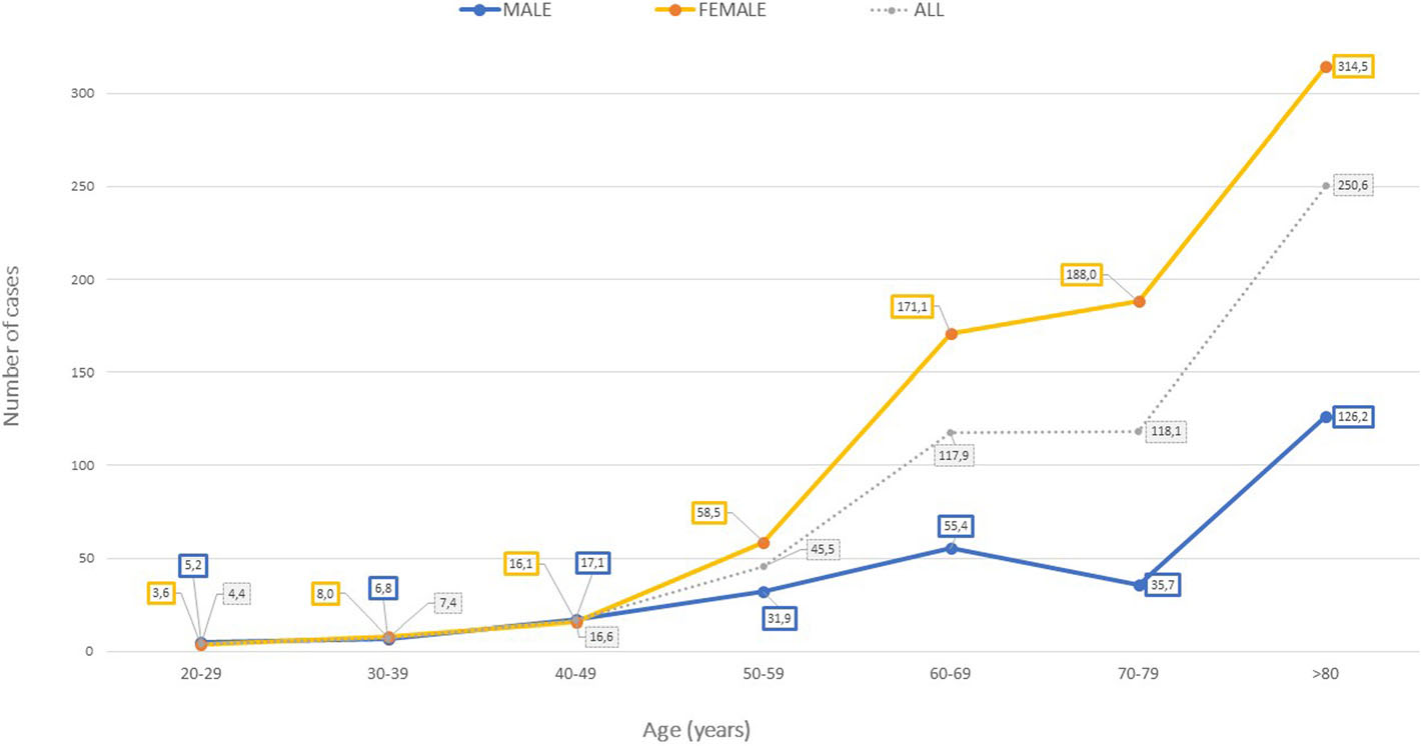
Distribution of the number of cases by age and sex

In the period studied, there were 7698 emergency room visits because of musculoskeletal conditions that were treated and followed up by the Upper Limb Unit of the CHUVI T&O department; 4319 of these were fractures (56.1%), which amounts to an annual incidence rate of 293.3 fractures per 100,000 inhabitants and year. As regards PHFs, we calculated an incidence rate of 60.1 fractures per 100,000 inhabitants and year. We calculated a gender-disaggregated incidence rate of 89.3 fractures per 100,000 women/year and 28.2 fractures per 100,00 men/year. The incidence distributed by decades is shown in Fig. 3 and Table 2.

**Table 2:** Annual incidence rates of humerus fractures (per 100,000 inhabitants-year) in our study in comparison with countries from different geographic regions of Europe

Author, year publication	Origin of study Number of patients (N)	Study period	Origin of patient database	Age of patients, years	Sex	Annual rate (× 10^5^ h/a)*	Our study 2016–2018 *N* = 638
Kristiansen et al. [8], 1987	Denmark *N* = 565	1983	Inpatients and outpatients	20–29	Female Male Female Male Female Male Female Male Female Male Female Male Female Male	4 2 16 33 61 33 111 32.5 208 98 359 115 502 182	**3.5** **5.1** **7.9** **6.8** **16** **17.8** **58.4** **31.8** **171** **55.4** **188** **35.7** **314.4** **126.1**
				30–39			
				40–49			
				50–59			
				60–69			
				70–79			
				> 80			
Lind et al. [18], 1989	Denmark *N* = 730	1980-1984	Inpatients and outpatients	≥ 18	All	73	**60.1**
Court-Brown et al. [10], 2001	Scotland *N* = 1027	1992-1996	Inpatients and outpatients	20–29	Female Male Female Male Female Male Female Male Female Male	4 5 4 15 20 20 45 28 96 34 188 58 260 109 139 159	**3.5** **5.1** **7.9** **6.8** **16** **17.8** **58.4** **31.8** **171** **55.4** **188** **35.7** ***(> 80)*** **314.4** ***(> 80)*** **126.1**
				30–39			
				40–49			
				50–59			
				60–69			
				70–79	Female Male Female Male Female Male		
				80–89			
				90–99			
Maravicz et al. [19], 2005	France *N* = 12262	2001	Inpatients	46–65	Female Male Female Male Female Male	23**.**5 17**.**3 107 29**.**8 247**.**5 78**.**5	**39.8** **28.8** **241.8** **95.1** **234.2** **126.1**
				66–80			
				> 80			
Palvanen et al. [5], 2006	Finland	1970-2002	Inpatients	≥ 60	All	32 ^(1970)^ 105 ^(2002)^	**148.9**
					Femal	51^(1970)^ 129 ^(2002)^	**215.4**
					Male	14^(1970)^ 48 ^(2002)^	**60.4**
Court-Brown and Caesar [2], 2006	Scotland *N* = 337	2000	Inpatients and outpatients	≥ 14	All	63	**60.1**
Péntek et al [7, 17], 2008	Hungary *N* = 41556	1999-2003	Inpatients and outpatients	65–69	All Female Male All Female Male	248 366 215 632 1151 632	**150.1** **188.1** **91.4** **214.1** **280.4** **106.2**
				80–84			
Dimai et al [9], 2013	Austria	1989-2008	Inpatients	≥ 50	Female Male	141 to 383 112 to 222	**166.7** **50.2**
Launonen et al. [3], 2015	Finland *N* = 678	2006-2010	Inpatients and outpatients	≥ 16 20–29	**All** Female Male Female Male Female Male Female Male	**82** 13 14 18 12 39 31 112 63 193 87 296 100 379 232	**60.1** **3.5** **5.1** **7.9** **6.8** **16** **17.8** **58.4** **31.8** **171** **55.4** **188** **35.7** **314.4** **126.1**
				30–39			
				40–49			
				50–59			
				60–69	Female Male Female Male Female Male		
				70–79			
				> 80			
Bergdahl et al. [1], 2016	Sweden *N* = 2011	2011-2013	Inpatients and outpatients	≥ 16	**All**	**83**	**60.1**
Sumrein et al. [20], 2017	Sweden *N* = 98770	2001-2012	Inpatients and outpatients	≥ 18	Female Male	134**.**5 ^(2001)^ 174**.**6 ^(2012)^ 68**.**1 ^(2001)^ 49**.**2 ^(2012)^	**89.3** **28.2**
Kannus et al. [21], 2017	Finland	1970 -2015	Inpatients	≥ 80	Female	87 ^(1970)^ 297 ^(2015)^	**314.4**
Klug et al. [22], 2019	Germany *N* = 642556	2007-2016	Inpatients	≥ 16	All	74**.**2	**60.1**

*(×105 h/a): per 100,000 inhabitants/year

## Discussion

In our study population, PHF fractures were the third most frequent in the upper limb after distal radius fractures (DRF) and metacarpal and phalangeal fractures and account for 14.7% of all fractures of this anatomical region and 8.2% of the global cause of emergencies due to upper limb pathologies during the period of study. In the article published in 2000 by Court-Brown and Caesar [4], with a population similar to ours, PHFs were 9.6% of upper limb fractures, in third place after distal radius fractures and metacarpal and phalangeal fractures.

One of the most interesting findings of our study is that the incidence rate observed in our sample (60.1 per 100,000 inhabitants/year) was lower than that published in other studies in Northern Europe [5, 18, 20, 22], but similar to that published in Scotland by Court-Brown and Caesar [4] (63 per 100,000 inhabitants/year) where patients treated both surgically and conservatively were included. These findings apply both for the analysis of the total sample and the segregation by age or gender and also includes both inpatients and outpatients.

We have also found variations in the incidence rate per age range referenced in other studies (Table 1). All these data might lead us to conclude that the PHF incidence rate may vary depending on the geographical area, activity, health, and care provided to the elderly, although the data from these studies cannot be compared rigorously. Regarding the age-adjusted incidence rate of our sample, it is most remarkable that higher values are observed with respect to the studies that only consider inpatients [7, 11, 19, 21, 22]. In contrast, the values in our sample were lower than the studies including both inpatients and outpatients [1, 4, 5, 10, 12, 18]. Of course, if a portion of the patients treated is not recorded, the incidence rate tends to be underestimated, and perhaps to an even greater extent today, since patients tend to be treated mainly on an outpatient basis.

In line with the articles published in the literature [1–13], we have observed a significant increase in the PHF incidence in people over 50 years old, especially in women (Fig. 1). Most of these fractures are due to low energy injuries, mainly due to falls from standing height. In our study, more than 50% of the population with PHF was over 70 years old, mostly women (84.2%). Up to 50 years of age, the female to male ratio was 1:1 and in people that were older than 50, the ratio became 4:1. This can be explained by the increased risk of falls in the older population, the unpredictability of the trauma, the greater likelihood to have osteoporosis, and by women’s greater life expectancy [3]. In 2010, Hernlund et al. [21] estimated that 54% of the total population of the European Union (EU) were women and that they had suffered two thirds of all accidental fractures. During the period of study, there was a similar distribution (53% women and 47% of men) in our healthcare area. Above 75 years of age, there were nearly 33% more women than men.

Similarly to other studies published [2, 3, 5], no statistically significant differences were found regarding the affected side, either analysing the total sample or segregating it by age or gender.

According to Roux et al. [2], there are two major risk factors for osteoporotic fractures and in particular for PHFs. The first risk is bone fragility followed by the risk of falling. The more fragile bones are, the more serious the fracture is [12]. In 2011, it was estimated that the prevalence of osteoporosis in the EU was 5.5% of the population, and in Spain, it was 5.4% (6.8% in men and 22.6% in women) [21]. In 2011, Calvo et al. [23] published a multicentre prospective study on 5147 postmenopausal women over the age of 50 with osteoporotic fractures in Spain. These authors concluded that PHFs accounted for 17.5% of the total number of osteoporotic fractures in this population and that only 1521 women (29.5%) took treatment for osteoporosis. In our study, 6.5% of patients with PHF (64 women and 1 man) were taking treatment for osteoporosis at the time they suffered a fall, and all of them were over 50. This accounts for 13.7% of women over the age of 50, which represents a significantly lower figure than the data published by Calvo et al. [24] and Hemlund et al. [21], although it is likely that this pathology is underdiagnosed in our population. Despite taking treatment for osteoporosis, three women, who fell on two separate occasions more than six months apart, suffered two PHFs each.

It has been described that cardiovascular diseases can be a factor favouring the occurrence of falls [2]. Just over half of the sample (51.7%) had cardiovascular disease, which is the most prevalent comorbidity in our study. Within that group, the cause of PHF in up to 98% of patients was a low-energy fall. Chu et al. [25] studied the risk factors associated to PHFs in patients older than 45 and concluded that falls, diabetes mellitus, and difficulty walking in low light are frequent aspects related to these types of fracture. However, Martinez-Huedo et al. [26] found that type-2 diabetes mellitus was a risk factor. In our study, 17.2% of patients with PHF were (type 1 and type 2) diabetic while the estimated percentage in the Spanish general population older than 18 years of age is 13.8%. It has also been suggested that epilepsy, depression, and dementia increase the risk of PHF. In our study, we have noted that 29.3% of the patients presented neurological or psychiatric diseases.

Almost all epidemiological studies showed seasonal variation in the frequency of PHF, with an increase of these fractures in winter [1–3, 5, 12, 18]. The authors justify these variations on the grounds of weather conditions (for example rain, wind, snow, mud, and ice) and of fewer hours of sunlight in winter, which can increase the risk of falls, car accidents, or both.

In this study, we found neither differences in distribution of PHF with respect to season nor a statistical correlation when considering trauma mechanism, age, or gender with regard to the season. These variables appear to remain stable, probably due to our moderate oceanic climate.

Regarding the type of injury that caused the high or low-energy PHF, we have found a correlation between injury mechanism and age. In fact, as observed in other studies [2, 18], there was a bimodal distribution depending on the mechanism of energy and the patient’s age (Fig. 3). Most high-energy injuries (65.2%) happened to patients under the age of 55, while in this age range, only 12.3% suffered low-energy trauma. This is justified because the younger population is more likely to engage in contact sports, has more intense physical activity, and drives cars or rides motorcycles. In contrast, it is usual that low-energy injuries happen to older patients, considering the progressive decrease in bone strength as a result of ageing.

We have also found a statistically significant association between the type of trauma suffered and the type of treatment. As much as 43.5% of patients with high-energy PHFs underwent surgical treatment, while only 18.7% of patients with low-energy trauma needed surgery. However, as in the study of Bergdahl et al. [1], there was no statistically significant association between the injury mechanism and the type of fracture according to the AO-OTA classification [14] or the Neer classification [15]. Two reasons could justify this: first, because 26% of patients who suffered high-energy trauma had another fracture associated in the ipsilateral limb that required surgical treatment, although not all of them had a complex proximal humerus fracture, and secondly, because older women who suffer a low-energy fall usually have complex fractures due to their poor bone quality.

The severity of fractures increases as people age [2]. In his article in 1970, Neer [15] estimated that approximately 85% of all PHFs were not displaced (type I) fractures. However, much lower percentages of these type-I fractures (from 13 to 49%) [3, 10, 11, 13, 18] have been found in recent studies, which is consistent with our data (24.3%). Even so, in the studies of Court-Brown et al. [12] and of Roux et al. [2], type I was the most frequent, while in other studies such as those by Launonen et al. [5], Lidn et al. [18], and Barhs et al. [13], type II prevailed. In our study, if we split the sample between younger and older than 50 years old, we find that type I fracture is the most common in both young male and female, while type II fracture is the most common in older male and type III in older female. With regard to more complex fractures (three and four parts, including fracture-dislocation), we have found a higher proportion than Court-Brown et al. [12], Launonen et al. [5], and Roux et al. [2], although it is similar to what has been published in the Swedish registry [20] (Table 1).

In reference to the AO-OTA classification [14], Court-Brown et al. [12] and Bahrs et al. [13] reported a higher percentage of type-A fractures (66% and 61%, respectively), while in the study of Bergdahl et al. [1], there was a similar percentage in type A and B and lower in type C (45%, 44%, and 11%, respectively). Type-A fractures were also the most frequent in our sample although the distribution was similar to what has been published by Bergdahl et al. [1] in their Swedish registry (49%, 43%, and 7.7%, respectively). Statistically significant differences between the different types were also found. These authors have postulated the greater percentage of elderly patients in their sample as a possible explanation, because type-A fractures are more frequent in young patients while type B and C fractures increase with age. This is in line with our study because type B and C fractures account for only 21.6% of PHFs in people under 50. Another possible explanation in this change of fracture pattern could be related to imaging techniques. We have done the initial assessment and radiographic review in this study with digital x-rays, which allow to enlarge images and to change contrast, so we have been able to detect fracture lines that may go unnoticed initially and, therefore, change the initial classification to type B or C fractures. However, it is important to keep in mind that these two classifications are subject to both wide interobserver and intraobserver variability [2].

Some important factors that influence decision making and the long-term clinical results in PHFs are the number of displaced parts and the patient’s age. The increase in the number of complex fractures, along with improvements in the surgical material with the appearance of locking plates and reverse shoulder arthroplasty, have led to a relative increase of surgical treatment of up to 40% [8, 23]. However, this trend has not been supported by studies of high scientific evidence and, in fact, there is considerable variation in current clinical practice. The Cochrane Library review published in 2015 showed no differences between the surgical and conservative treatment for complex PHFs [23]. No statistically or clinically significant differences were found between the surgical and non-surgical treatment in the ProFHER randomised clinical trial [27] in patients with displaced PHFs or in the systematic review and meta-analysis carried out in 2015 by Rabbi et al. [28]. In our study, 75.7% of PHFs were displaced fractures. However, only 25% were treated surgically (20% of Neer type II and 28% of the group of three or more parts, including fractures-dislocations). This lack of correlation shows how difficult it is to handle this type of patients, because of the bone quality and comorbidities in elderly patients. More than 40% of the patients in our study were older than 75 at the time of the fracture and more than 95% of these patients had some kind of associated pathology.

Like in other studies [20, 22], the most used surgical treatment was locking plates (47 cases) especially for 2-part and 3-part fractures, followed by reverse total shoulder arthroplasty (36 cases), which was used most frequently in our study for treatment of 4-part PHFs. As we have mentioned before, the use of these treatments has increased rapidly in comparison to others, although it has not been possible to demonstrate that they are clearly better approaches [5, 23].

### Limitations and strengths of the study

This study presents some limitations. The retrospective nature and the relatively small population in the study are a limitation, as well as the fact that the classification of fractures was carried out by five orthopaedic surgeons, which further increases the likelihood of finding high interobserver variability. As strengths of this study, we have been limited to the registration of one hospital where all the emergencies and urgencies of the above-mentioned metropolitan area are treated, since it is accessible to the whole population because of the comprehensive healthcare system in Spain. Another strong point is that we have been able to collect all the PHFs that occurred in the above-mentioned area, including patients and outpatients and both surgical and non-surgical treatment. Due to these two strengths, we believe we have selected a representative sample of our region, and it might be extrapolated to the rest of the Spanish population for the epidemiological analysis of this pathology. Furthermore, no similar epidemiological studies have been published in Southern-Mediterranean European countries, which share similar lifestyle, nourishment, climate conditions, and life expectancy. Our results could resonate better with the epidemiology of PHF in these countries in comparison with northern and central European registries. However, besides this fact, comparisons with other studies should be made with caution, especially due to differences in the criteria for patient selection.

## Conclusions

PHFs are a very frequent pathology, and they are the third most frequent upper-limb fractures, without seasonal variability in our area (temperate oceanic climate).

Older women who suffer a low-energy mechanism are the most affected.

Most fractures are displaced, and the number of complex fractures increases with the age of the population.

There has been an increase in the surgical treatment for these fractures in recent years, but it is not in accordance with the increase of complex fractures.

It is important to adopt measures in the ageing population in order to prevent PHF risk factors from becoming a new source of dependency for the elderly population.

## Data Availability

The datasets used and/or analysed during the current study are available from the corresponding author on reasonable request
